# Intelligence

**Published:** 1831-03

**Authors:** 


### INTELLIGENCE.

Botanical Lectures. Mr. G. T. Burnett, Professor of Botany to the Medical Botanical Society, &c., will commence, early in March, an extended and practical series of lectures on Botany; to be delivered in the School of Physic, St. George's Hospital.

---

Medical Degrees *at the* London University *and* King's College. It has been erroneously reported, that the power of conferring medical degrees has been given to these institutions. It is probable that this important privilege may ultimately be conferred, but as yet no definite arrangement has been made upon the subject.

---

Medical School, Aldersgate street. The anniversary dinner of this establishment took place on the 18th February, at the London Coffeehouse, Ludgate hill; on which occasion, Mr. Quain took the chair. The company assembled was numerous; an excellent dinner was served up, and the utmost conviviality prevailed during the evening. The affairs of the institution appear to be in a very flourishing condition ; and it was pleasing to notice the very strong feelings of goodwill which existed between the teachers and pupils, or, as the former express themselves, between seniors and juniors, it being their anxious wish to do away with that cold formality which is sometimes observed to take place between the lecturers and their classes.

---

Medical Witnesses. It has recently been determined, in the Court of King's Bench, that a medical practitioner cannot claim any remuneration for the time taken up in attending trials at law, upon which he has been subpœned as a witness.

---

*To the Editor of the London Medical and Physical Journal.*

Sir: Will you permit me to inquire, through the medium of your Journal, whether any well-authenticated instance exists of a man being enabled to withdraw his testicles from the scrotum, within the abdominal ring, at his own pleasure? The reason why this inquiry is made, is, that a case has lately occurred, and is now under legal investigation, in which the different circumstances stated in evidence cannot at all be reconciled with each other, unless the accused person possesses this power. But, if so, much at present contradictory evidence will be reconciled, and a witness, of hitherto good reputation, be rescued from the imputation of perjury. Under these circumstances, I trust that you will insert this inquiry in your next number, an early answer being desirable. If any of your correspondents have had any instance of the kind occur, in the course of their practice, they are also desired to state the particulars.

I remain, sir, yours, &c.

LEGALIS.

22*d January*, 1831.

The best evidence with which we can furnish Legalis upon the point in question, is taken from "Marshall's Hints to Medical Officers," p. 169. If any of our readers have met with similar cases, we shall be happy to publish an account of them.

" Hernia. This infirmity has been simulated by persons who possessed the power of drawing the testes up to the rings of the external oblique muscles, availing themselves of this power to deceive medical officers. I have seen a number of individuals, who possessed this faculty, some being able to elevate the testicle of one side, but not the other. According to Baron Percy, there are individuals who can voluntarily retract a testicle within the abdomen. The subject of the following case seems to have possessed this power: Pat. Gafney enlisted in the second battalion of the rifle brigade, in 1825. Gafney subsequently reported that he was ruptured. He was examined by Dr. Connell, who found a slight degree of fulness over the left inguinal ring. On prosecuting the examination, he ascertained that the left testicle was not in the scrotum: by means of considerable pressure, applied immediately above the ring, the testicle was excluded; he continued, however, for some time to exert the voluntary power he possessed over the cremaster muscle, which was considerable, to elevate the testicle. Before Gafney enlisted in the rifle brigade, he had served several years in the marines, from which corps he was discharged in consequence of inguinal hernia, the result of service, with a pension. This fact was discovered shortly after he enlisted, by his coming to the orderly room with his instructions, and shewing them to the clerk, informing him, at the same time, that the rupture was cured. He was discharged from the army, with ignominy, in September 1827."

We are informed, that a similar attempt at imposition was once made by a sailor, who was examined by Mr. Copland Hutchinson: Mr. H., however, discovered the trick.—Editor.

### COLLEGE OF PHYSICIANS.

### Sir Henry Halford *on the Influence of some of the Diseases of the Body on the Mind.*

The first meeting of the season, at the College of Physicians, took place on Tuesday evening, February 1st: it was attended by a large number of men of science, and distinguished members of the three learned professions. Among them were, the Lord Chancellor, the Archbishop of Canterbury, the Bishop of London, Lord Tenterden, the Vice Chancellor, the Master of the Rolls, Mr. Baron Vaughan, several of the Judges, the Attorney General, &c. &c.

Sir Henry Halford took the chair, as president, and proceeded to read a paper, which was listened to with the deepest attention, and of which we subjoin an account. The essay had for its object to portray the influence of diseases of the body on the mind, and to discuss certain questions relating to the duties of the physician immediately connected with this subject.

The effects of the passions on the body have been elegantly illustrated by Sir G. Baker; nor is the converse of the picture less interesting. The influence of disease on the mental faculties is various and extensive; now communicating "temporary power," and now "permanent weakness;" while so characteristic are some of these changes, that the physician is often able to discover, by attention to these secondary effects, what organ is at fault, without calling into play all those appliances which are usually adopted, in order that the whole case may be fully unfolded. Nor are the diseased conditions at all proportionate in danger to the degree of mental affection which they produce: a simple derangement of the digestive organs lays a weight upon the mind, rendering the individual irresolute and infirm of purpose; whilst an inflammation of the brain, if it be slight, gives intensity to his faculties and animation to his imaginings.

Sir Henry stated it to be his intention to give only an outline of the effects upon the mind produced by some of the more marked and simple chronic diseases, leaving it to his hearers to fill up the details from their own experience.

Of apoplexy, little was to be said, because, before the attack, the patient is generally merely torpid, and, after it has occurred, is insensible to all around; so that, however momentous or interesting to others may be passing events, "nothing touches him." To apoplexy succeeds palsy, and happy he (said the learned President,) "whose mind shall have been disciplined when in health, and whose moral habits shall have been well regulated by reason and by good principles before he was taken ill; for, otherwise, as all the passions are let loose by the malady, (at least, this is the case in many instances of palsy,) whilst the controlling power is enfeebled, an irritability succeeds, which makes life intolerable to the sick man himself, and to all around him." The picture of this melancholy state was further coloured by vivid description, and illustrated by reference to the cases of the Duke of Marlborough and Dean Swift.

> "From Marlbro's eyes the tears of dotage flow,
> And Swift expires a driveller and a show."

Epilepsy, so much the terror of others, carries not mental suffering to the patient himself: he sleeps after the paroxysm, and awakes unconscious of what has passed. Repetition of the fits, however, weakens the intellect, and ultimately leads the way, in many cases, to madness or fatuity. This, however, does not apply to epilepsy which is merely sympathetic of derangement of the alimentary canal, or the forerunner of some eruptive disease, or other transient derangement. In this way were Julius Cæsar and Mahomet epileptics. In pulmonary consumption, the frame is often lighted up, and every thing looks cheering to the patient, where the friends and medical attendant see the prospect inevitably ending in the tomb. The frame of mind in a hectic girl, with all the buoyancy of youth, was then contrasted with that of a female more advanced in life,—at the period when the menses cease: the former all hope, and the latter all despondency. This contrast the pathologist might, perhaps, attempt to explain by referring to the different conditions of the circulation in the two. The blood in the younger may be more oxygenated, from its more rapid circulation through the lungs; while, in the elder, there is a stagnation about the vena portæ, giving rise to hypochondriasis, and connected with the more loaded state of the ventral and hemorrhoidal veins, from the cessation of the menstrual discharge.

The learned President next adverted to those cases in which the heart and great vessels were diseased. In these the paroxysms are dreadful, but the intervals are comparatively tranquil, like the calm which succeeds the tempest. Such patients, indeed, are "full of life." and often are much less dejected than those who suffer only from derangement of the stomach. Their condition illustrates the observation of PALEY, with respect to pain, "that its pauses and intermissions become positive pleasures; that it has a power of shedding a satisfaction over the intervals of ease, which few enjoyments exceed." That mere pain does not affect the faculties to any great extent, is also manifested by what we witness in tic douloureux and iliac passion; in which last, many days and nights of misery frequently are insufficient to impair the judgment of the patient, or destroy his hopes. "From such sufferings as these," added Sir Henry, nearly in the following words, "death may well be considered a happy release: indeed, before the glad tidings of pardon and peace in a future life, on certain conditions, had been proclaimed to the world by our Redeemer, so much intense suffering, nay, much less than that which is endured by a patient under fatal ileus, was considered by the most enlightened Romans as a sufficient reason for ridding themselves abruptly of life. The first book of Pliny's Letters furnishes us with two instances of friends of his, one of whom had recourse to this apparently common practice, and the other intended to resort to it if the physician should pronounce his malady a mortal one. Their creed admitted an independent exercise of their free will and pleasure in the disposal of their lives. "Ipse deus, simul atque volam, me solvet; moriar, mors ultima linea rerum est." But the Christian has a higher motive for submitting himself to the will of Heaven, and for bearing his sufferings patiently.

Upon the whole, Sir Henry has been astonished that so few of the great numbers to whom he has ministered in their last hour, have been reluctant to part with life. Some, indeed, have clung to it with painful anxiety; but this has been more frequently from the agony of leaving their children to the mercy of the world, than from mere love of life. This part of the subject led the learned author of the paper to speak of the duty of the physician, in withholding from, or communicating to, a patient the probable issue of a disease displaying mortal symptoms. He stated it to be his opinion that the first duty of the physician was "to protract the life of his patient by all practicable means," to interpose between him and whatever was calculated to aggravate his danger; and that, unless the patient should be averse from doing what is necessary in aid of the remedies, owing to the want of a proper sense of his danger, the medical attendant ought not to step out of his province to offer advice: at the same time, he held it to be indispensable that the friends be informed of the danger the instant it is discovered. It is far better that friends should undertake the delicate task of suggesting to the patient, at proper times and under the guidance of the physician, the necessity of arranging his worldly affairs, and preparing for his momentous change. But there may be no friends present, no one near him, from whom the intimation of his danger would come with propriety: under such circumstances, Sir Henry said that he should feel warranted in departing from his strict professional duty, and apprising the patient of his condition.

The part of the paper which followed was listened to with peculiar interest, from the direct relation it bore to the case of his late Majesty, as well as from other circumstances connected with some of the eminent individuals who were present. If discretion be required by the practitioner with regard to cases attended with danger in private life, this becomes doubly requisite in a personage "of a station so elevated, that his life becomes an object of anxiety to the nation." The public, in their solicitude for the recovery of such a patient, frequently desire disclosures incompatible with his safety. The bulletins may become known to the royal sufferer himself; and it cannot be admitted for a moment, that, to relieve the anxiety or gratify the curiosity of the public, the physician ought to do any thing to endanger the life or comfort of his patient. "But," added Sir Henry, "whilst it is his object to state as accurately as possible the present circumstances and the comparative condition of the disease, he will consider, that conjectures respecting its cause and probable issue are not to be hazarded without extreme caution. He will not write one word which is calculated to mislead: but neither ought he to be called upon to express so much as, if reported to the patient, would destroy all hope, and hasten that catastrophe which it is his duty, and their first wish, to prevent. Meantime the family of the monarch, and the government, have a claim to further information than can with propriety, or common humanity, be imparted to the public at large. In the case of his late Majesty, the king's government and the royal family were apprised, as early as the 27th of April,* [Sir Henry held in his hand the original letters which gave information to the prime minister,] that his Majesty's disease was seated in his heart, and that an effusion of water into the chest was soon to be apprehended." It was not until the end of May that an opportunity occurred of acquainting his Majesty with his real situation: he then appointed an early day for receiving the sacrament, and expressed himself as having derived great consolation from this exercise of

* The late king died on the 25th June.

devotion. After this, Sir Henry thought himself warranted in interpreting the symptoms as favorably as they would admit, and was thus enabled to rally the spirits of his royal patient in the intervals of his suffering, and prevent him from dwelling on the painful contemplation of death until a few minutes before he expired.

### MONTHLY LIST OF MEDICAL BOOKS.

### METEOROLOGICAL JOURNAL.

**By Messrs. Harris and Co. Mathematical Instrument Makers, 50, High Holborn.**

| *Month.* | *Moon.* | *Rain gauge.* | *Therm.* 9 A.M. | *Therm.* MAX. | *Therm.* MIN. | *Barom.* 9 A.M. | *Barom.* 10 P.M. | *De Luc's Hygrom.* 9 A.M. | *De Luc's Hygrom.* 10 P.M. | *Winds.* 9 A.M. | *Winds.* 10 P.M. | *Atmospheric Variation.* 9 A.M. | *Atmospheric Variation.* 2 P.M. | *Atmospheric Variation.* 10 P.M. |
|---|---|---|---|---|---|---|---|---|---|---|---|---|---|---|
| Jan. | | | | | | | | | | | | | | |
| 20 | | | 44 | 47 | 39 | 29.56 | 29.48 | 75 | 75 | SSW | S | F.&Sl. | Sleet | Cloudy |
| 21 | ☽ | | 42 | 45 | 40 | .30 | .07 | 75 | 76 | E *va.* | SE | Foggy | Cloudy | Sm. R. |
| 22 | | | 44 | 49 | 44 | .00 | .02 | 78 | 79 | ESE | SSE | Sl.&F. | Foggy | Cloudy |
| 23 | | | 47 | 49 | 36 | .07 | .08 | 80 | 80 | SE | SSE | Foggy | — | — |
| 24 | | | 41 | 48 | 33 | .21 | .27 | 80 | 79 | SE | N | — | Sho'ry | — |
| 25 | | | 35 | 34 | 31 | .35 | .42 | 79 | 74 | N | N | Snow | Cloudy | Fine |
| 26 | | | 33 | 34 | 26 | .64 | .84 | 69 | 66 | NNW | N | Fine | Fine | Fine |
| 27 | | | 30 | 32 | 27 | .95 | 30.05 | 64 | 64 | N | NNW | Fine | — | — |
| 28 | ○ | | 32 | 37 | 31 | .74 | 29.23 | 65 | 66 | WSW | W | Foggy | Rain | Rain |
| 29 | | | 34 | 34 | 30 | .51 | .55 | 67 | 66 | NNW | NW | Fine | Fine | Fine |
| 30 | | | 32 | 32 | 29 | .75 | .71 | 63 | 62 | N | S | — | — | Cloudy |
| 31 | | | 31 | 33 | 27 | .68 | .62 | 62 | 62 | SE | SE | — | — | Foggy |
| Feb. | | | | | | | | | | | | | | |
| 1 | | | 30 | 33 | 29 | .58 | .43 | 62 | 66 | SW | S | Foggy | Foggy | Snow |
| 2 | | | 31 | 34 | 20 | .97 | .52 | 70 | 70 | S | SW | Snow | Cloudy | Fine |
| 3 | | | 33 | 35 | 29 | .02 | .07 | 70 | 70 | SSW | SSW | Cloudy | S.& R. | Fine |
| 4 | ☾ | | 32 | 42 | 32 | .27 | .00 | 70 | 74 | SSW | SE | Fog | Rain | Sho'ry |
| 5 | | | 43 | 43 | 30 | 28.85 | .26 | 78 | 76 | S | W *va.* | F.&R. | Cloudy | Fine |
| 6 | | .17 | 38 | 43 | 29 | 29.56 | .75 | 70 | 70 | NW *v.* | NW | Fine | Fine | — |
| 7 | | | 31 | 33 | 37 | .64 | .48 | 71 | 73 | N | SE | — | — | S.& R. |
| 8 | | | 40 | 44 | 46 | .36 | .61 | 73 | 75 | SW | SW | Cloudy | Fine | Rain |
| 9 | | | 50 | 53 | 53 | .72 | .78 | 75 | 74 | SW | SW | Cloudy | Rain | Cloudy |
| 10 | | .13 | 55 | 59 | 51 | .85 | .95 | 63 | 60 | SW | WSW | Fine | Fine | Fine |
| 11 | | | 55 | 58 | 50 | 30.07 | 30.07 | 56 | 58 | SSW | SW | — | — | — |
| 12 | ● | | 55 | 56 | 50 | .08 | .11 | 59 | 60 | WSW | SW | — | — | Cloudy |
| 13 | | | 51 | 50 | 48 | .13 | .13 | 62 | 63 | WNW | SSW | Cloudy | — | — |
| 14 | | | 50 | 50 | 46 | .08 | .06 | 65 | 66 | S | SW | Sho'ry | Sho'ry | — |
| 15 | | .03 | 48 | 50 | 41 | .08 | .03 | 67 | 66 | SW | SSW | Cloudy | Fine | Fine |
| 16 | | | 43 | 47 | 42 | 29.96 | 29.77 | 63 | 63 | SE | S | Fog | — | — |
| 17 | | | 46 | 52 | 42 | .77 | .90 | 62 | 62 | WSW | WNW | Fine | Cloudy | — |
| 18 | | | 45 | 49 | 38 | .86 | 30.01 | 62 | 61 | NW | NW | Sho'ry | Fine | — |
| 19 | ☽ | | 46 | 47 | 40 | 30.04 | .03 | 61 | 63 | W | W | Cloudy | — | — |

The quantity of Rain cannot be given for January, owing to severe frost, and the Rain Gauge not being exposed.

